# From the Ground Up: Can Root Traits Help Understand Invasion Dynamics?

**DOI:** 10.1002/ece3.74107

**Published:** 2026-07-31

**Authors:** Amoi Campbell, Jennifer L. Funk, Sara Kuebbing, Martin A. Nuñez, Gabriela C. Nunez‐Mir, Matthew A. McCary

**Affiliations:** ^1^ Department of Biosciences Rice University Houston Texas USA; ^2^ Department of Plant Sciences University of California Davis California USA; ^3^ The Forest School at the Yale School of the Environment Yale University New Haven Connecticut USA; ^4^ Department of Biology and Biochemistry University of Houston Houston Texas USA; ^5^ Department of Biological Sciences University of Illinois Chicago Illinois USA

**Keywords:** biodiversity, community, disturbance, invasibility, invasion biology, nutrient availability, risk assessment

## Abstract

Trait‐based comparisons of invasive and native plants have advanced our understanding of invasion mechanisms, but most focus on aboveground traits, overlooking the influence of root traits. In this mini‐review, we synthesize existing research to evaluate the often‐neglected role of root traits in plant invasions across different levels of disturbance and nutrient availability. We then propose a new Root Trait–Environment–Invasion (RTEI) framework that accounts for how environmental context interacts with root traits to mediate invasion success. Our review suggests that there is no single root trait that correlates with invasion. However, we did identify emerging patterns that varied with the level of disturbance and nutrient availability. For example, in low‐disturbance, high‐nutrient environments, we found that plant invaders often display root traits associated with enhanced resource acquisition, such as higher root biomass. In contrast, invasive plants generally exhibited more fine root traits, such as increased specific root length than native plants in high‐disturbance, low‐nutrient environments. From the synthesized literature, we derived the RTEI framework, which predicts that acquisitive root traits associated with enhanced nutrient uptake will promote invasion success under high‐nutrient, low‐disturbance conditions. In high‐disturbance environments with abundant nutrients, invaders are expected to exhibit traits enabling rapid root turnover or clonal regeneration. In low‐nutrient environments, traits that foster microbial mutualisms or resource conservation may enhance invasiveness. *Synthesis*. By integrating root traits into invasion science, the RTEI framework offers a novel lens for predicting invasions across environmental contexts, thereby broadening invasion ecology to more fully account for belowground processes.

## Graphical abstract

Although root traits are known to influence invasion success, few studies have examined how invader roots differ from those of native plants. In this mini‐review, we present the Root Trait–Environment Invasion (RTEI) framework, which outlines hypotheses for how invader root traits differ from native species across disturbance regimes and nutrient conditions.

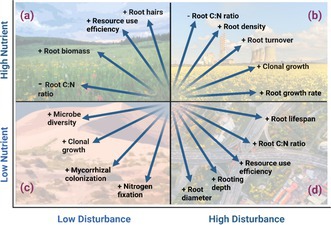

## 1 Introduction

Understanding why some nonnative plants become invasive while others fail remains a central challenge in ecology (Divíšek et al. 2018; Richardson et al. 2000). At its core, invasion success is determined by a species' ability to acquire resources (Davis et al. 2000; Gioria and Osborne 2014), tolerate environmental stress (Alpert et al. 2000; Chakraborty and Li 2010), and outcompete resident species (Carboni et al. 2021; Gioria et al. 2023); such processes are heavily governed by interactions that occur belowground (Broadbent et al. 2018; Levine et al. 2003). Plant roots are critically involved in these processes by regulating nutrient and water uptake in ways that mediate competitive interactions and plant–soil feedbacks (Bais et al. 2006; Bardgett et al. 2014; Foxx and Fort 2019). Consequently, root traits can strongly influence plant performance, population persistence, and community composition (Klimešová et al. 2018; Nunez‐Mir and McCary 2024; Ottaviani et al. 2020). In recent years, interest in understanding how roots influence plant community dynamics has grown considerably, as evidenced by the development of databases like the Global Root Trait Database (Guerrero‐Ramírez et al. 2021), Fine‐Root Ecology Database (Iversen et al. 2017), and the inclusion of belowground traits in the TRY Plant Trait Database (Kattge et al. 2011). However, researchers have been slow to incorporate this knowledge into invasion ecology, limiting our ability to identify and predict future invaders.

Variation in root traits (e.g., root tissue density, depth, specific root length, etc.) can predict plant performance under nutrient limitation (Freschet et al. 2010; Kramer‐Walter et al. 2016), with the potential to influence competitive hierarchies among co‐occurring species (Fort et al. 2013) and contribute to shifts in plant community composition (Bardgett et al. 2014). These traits define major axes of plant strategies belowground (Bergmann et al. 2020) and shape ecological processes across scales, from individual performance to ecosystem functioning (Valverde‐Barrantes et al. 2013). However, invasion ecology has only partially integrated this progress. Instead, most trait‐based invasion research has emphasized the importance of aboveground traits, such as leaf area, biomass allocation, and growth rate (Matzek 2012; Montesinos 2022; Van Kleunen et al. 2010). While such studies have been instrumental in advancing our understanding of invasive plant performance, these traits explain only a fraction of invasion outcomes and often ignore belowground patterns (Thompson and Davis 2011).

The competitive advantage conferred by particular root traits likely depends on the abiotic and biotic environment in which they are expressed (Laughlin 2014). Two environmental factors that are especially relevant are disturbance regimes and nutrient availability. Disturbance can strongly restructure soil biophysical conditions and competitive hierarchies (Fraterrigo and Rusak 2008; Nash Suding and Goldberg 2001), while nutrient enrichment shifts selection along the acquisitive–conservative spectrum of belowground strategies (de la Riva et al. 2021; McCormack and Iversen 2019). These two environmental drivers, disturbance and nutrient availability, can modify plant fitness on the landscape, altering which root traits are favored and when nonnative species can gain a competitive advantage. This environmental contingency highlights the need for trait‐based frameworks that explicitly account for how disturbance and nutrient availability filter root strategies during plant invasion.

Here, we synthesize existing research to explore how root traits correspond to invasion patterns in relation to disturbance and soil nutrient availability. We then introduce a new framework that connects root traits to invasion outcomes across ecosystems and propose testable hypotheses to guide future empirical research. By explicitly integrating belowground traits into invasion frameworks, we can gain deeper insight into the mechanisms driving invasion dynamics. Our goals are to (1) highlight and synthesize how root traits contribute to invasion success across gradients of disturbance and nutrient availability and (2) propose new directions for using root trait research to advance our understanding of invasion ecology.

## 2 Root Trait–Environment–Invasion (RTEI) Framework

Our Root Trait–Environment–Invasion (RTEI) framework considers how root traits associate with invasion patterns across varying disturbance and soil nutrient levels. The disturbance axis reflects the intensity of environmental disruptions, such as drought or habitat destruction, where high disturbance represents environments with a higher frequency or magnitude of disturbance than low disturbance. The nutrient axis is characterized by soil nutrient availability that ranges from low to high.

We focus on disturbance and nutrient availability because they represent two tractable, broadly applicable belowground filters that shape root trait selection and have been proposed as major drivers of invasion success in previous research (Alpert et al. 2000; Funk 2013). In doing so, we emphasize invader–environment interactions, complementing the extensive body of work on soil biota, plant competition, and plant–soil feedbacks (Dawson 2015; Reinhart and Callaway 2006; van der Putten et al. 2013). Disturbance and soil resource availability are also among the best‐studied environmental drivers of invasion, making them well‐suited for synthesis despite the limited number of studies that explicitly examine root traits. Although other biotic processes undoubtedly influence invasion outcomes, they fall outside the scope of this review and have been comprehensively synthesized elsewhere (see recent reviews by Negesse et al. 2025; Torres et al. 2021; Xu et al. 2022). Our goal is to highlight how environmental context shapes the relationship between root traits and plant invasion.

### 2.1 Disturbance Axis

Environmental disturbances are well‐known to influence the invasibility of native plant communities (Alpert et al. 2000; Kowarik 1995), and some nonnative plants can only invade a community after a disturbance has occurred (Alpert et al. 2000). Here, disturbance represents a diverse array of ecological stressors that vary in intensity, frequency, and mode of action. Despite these differences, disturbances generally modify soil physical and chemical properties, alter resource availability, reduce biotic resistance, and create opportunities for colonization by forming newly available space. These common effects have long been recognized as key mechanisms promoting plant invasion (Davis et al. 2000; Hobbs and Huenneke 1992), making it useful to consider disturbance as a single environmental axis in this review.

Because disturbances vary in type, intensity, and frequency, they impose distinct selective pressures that favor different root trait strategies in invading plants. Following disturbance, traits that promote rapid root proliferation, clonal regeneration, and efficient post‐disturbance resource capture are likely to enhance establishment and spread. In contrast, disturbance suppression (e.g., fire exclusion) might favor deeply rooted or highly competitive invaders (Grace and Zouhar 2008). Because disturbances often alter soil resource availability, either increasing or decreasing it depending on ecological context (Davis et al. 2000), they act as important environmental filters that influence both plant invasion dynamics and root trait expression.

### 2.2 Nutrient Availability Axis

Variation in nutrient availability is another fundamental driver of plant performance and community assembly, and invasion success is often related to accessing limited soil resources (Davis et al. 2000; Shea and Chesson 2002). Although resources include water, light, and space, we focus specifically on soil nutrient availability because it is a primary axis along which belowground acquisitive–conservative trade‐offs are expressed (Bergmann et al. 2020). Moreover, soil nutrient availability represents a continuous environmental gradient that can vary independently of physical disturbance through processes such as soil development, fertilization, nitrogen deposition, and land‐use legacies.

Root traits determine how plants acquire and conserve limiting nutrients in the soil. In low‐resource systems, invasive species may succeed when their root traits promote efficient nutrient scavenging, high nutrient‐use efficiency, nitrogen fixation, or recruitment of microbial symbionts (Richardson et al. 2000). Conversely, nutrient enrichment via fertilizer runoff or atmospheric deposition can favor fast‐growing, acquisitive root strategies that amplify competitive asymmetries (Broadbent et al. 2018; Dukes et al. 2011). Because nutrient availability can shift independently of mortality or biomass removal, it constitutes a distinct axis of environmental filtering that acts directly on root economic strategies.

### 2.3 Disturbance and Nutrient Availability Interactions

Disturbance and nutrient availability often interact in shaping invasion dynamics (Davis et al. 2000; Sher and Hyatt 1999). Disturbances frequently modify soil resource availability, either increasing or decreasing it depending on ecosystem context and disturbance type, while simultaneously imposing direct mortality and structural disruption (Burton et al. 2020; Pickett et al. 1989). Rather than treating these forces as independent, we conceptualize them as complementary, interacting axes that jointly structure the selection pressures on plant invaders belowground. Considering both dimensions concurrently allows us to distinguish mortality‐based filters from resource‐supply filters and evaluate how their interaction may favor different root functional strategies. Additionally, because both abiotic and biotic root interactions can shift along these gradients, the adaptive value of particular root traits, and even the direction of selection acting on them, may vary across disturbance regimes and levels of resource availability. Thus, these interacting axes provide a simple yet mechanistically grounded framework for understanding how root functional strategies differ between native and invasive plants across environmental contexts.

## 3 Systematic Literature Search

To review how root traits differ between native and invasive plants across gradients of disturbance and nutrient availability, we conducted a systematic literature search in ISI Web of Science (February 2023) using the following search terms without restriction on year: (invasive plant* OR exotic plant* nonnative weed* OR noxious weed* OR invasive weed* OR alien plant*) AND (disturb* OR fire* OR burn* OR prescribed burn* OR drought* OR flood* OR abandon* OR agriculture* OR urban* OR city) AND (nutrient availability* OR high resource* OR low resource* OR nitrogen availability* OR phosphorus availability*) AND (root trait* or belowground trait* belowground competition). The initial search identified 108 published articles, with 117 additional studies discovered through reference lists and by reviewing the publication records of researchers specializing in root traits and plant invasion. We then evaluated each article's title and abstract to determine whether it was eligible for inclusion in the systematic review (Appendix S1: Tables S1–S3); we used no unpublished datasets in this study. We followed the Preferred Reporting Items for Systematic Reviews and Meta‐analysis (PRISMA; Moher et al. 2009) search protocol to conduct the literature search and paper screening (Appendix S2: Figure S1). Our final search yielded 22 articles.

We evaluated papers that included any potential root trait, but we only uncovered ten traits that fit our criteria across the literature (Appendix S2: Table S1): (1) root nitrogen uptake, (2) nitrogen use efficiency, (3) root‐to‐shoot ratio, (4) root biomass, (5) root density, (6) root diameter, (7) nitrogen content, (8) root surface area, (9) root tissue density, and (10) specific root length. Additional inclusion criteria required that studies report trait data for both invasive and native species and clearly define the environmental context. We excluded reviews, modeling studies, and meta‐analyses. Each study was assigned to one of four scenarios based on relative levels of disturbance and nutrient availability, with criteria for disturbance and nutrient levels based on author‐defined experimental treatments. Full search methods are provided in Appendix S2.

Because we did not find sufficient articles to estimate effect sizes using a standard meta‐analytic model, we instead employed a vote‐count meta‐analysis (Bushman and Wang 2009). This type of meta‐analysis codes for three outcomes: (1) a positive significant statistical test, (2) a negative significant statistical test, and (3) no statistical difference. Here, we coded a positive outcome when the invasive plant species had a significantly higher value for a root trait compared to the native plant (i.e., *p* < 0.05 for an invasive plant having a *higher* trait value); a negative outcome was assigned when the native plant had a significantly higher trait value than the invasive plant (i.e., *p* < 0.05 for an invasive plant having a *lower* trait value). A mixed outcome was assigned if there was no statistically significant difference between the invasive and native plants (i.e., *p* > 0.05). All trait values in the vote count were characterized this way and then summed across all the studies for each combination of disturbance and nutrient availability. Although this form of meta‐analysis does not calculate an effect size to determine magnitudes of effects, it adequately summarizes findings across studies within a specified discipline (Borenstein et al. 2009).

## 4 Review and Synthesis

**FIGURE 1:**
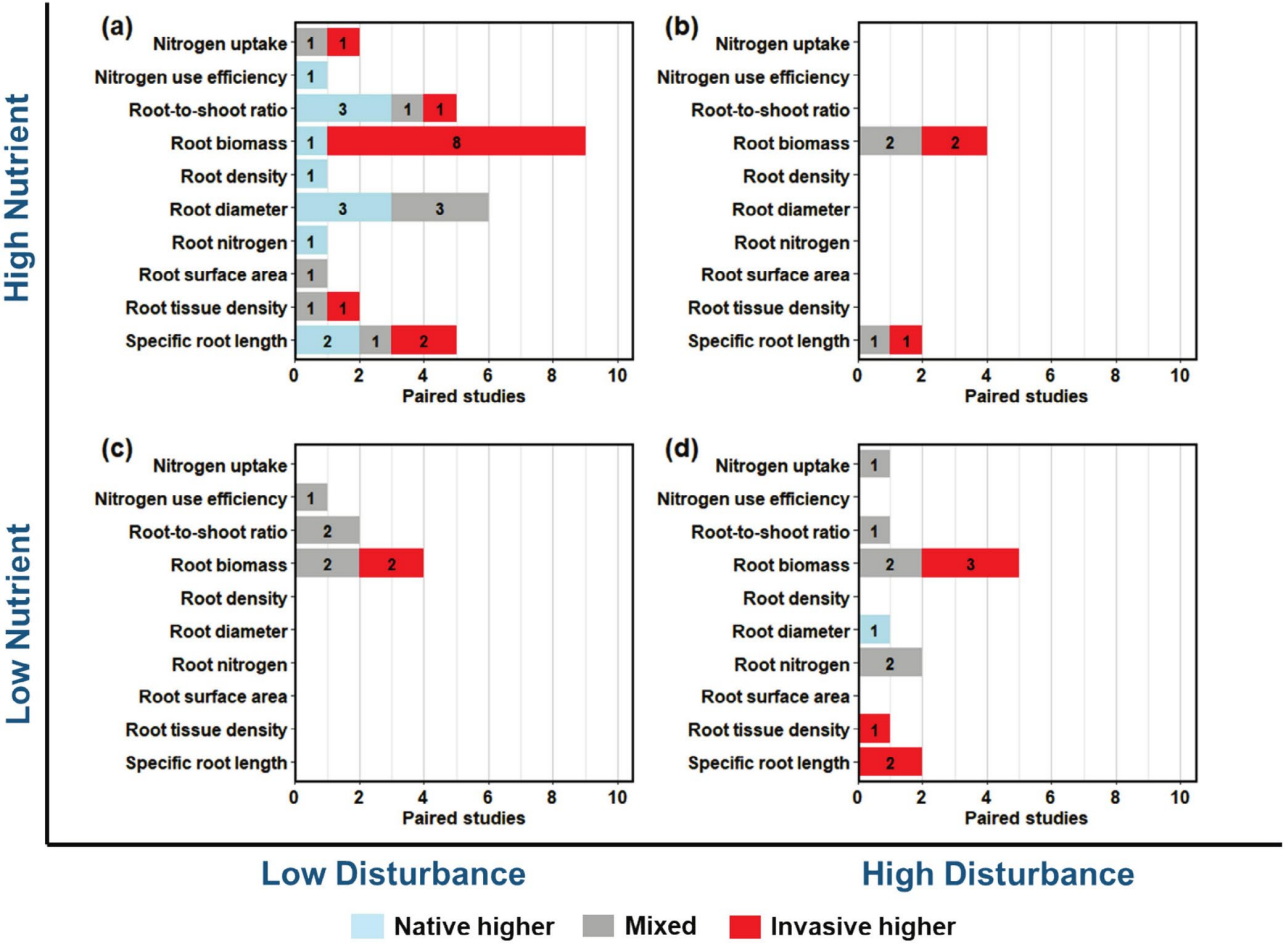
Root traits represented across four environmental scenarios: (a) low‐disturbance and high‐nutrient availability, (b) high‐disturbance and high‐nutrient availability, (c) low‐disturbance and low‐nutrient availability, and (d) high‐disturbance and low‐nutrient availability. Each scenario is a stacked bar plot showing the number of invasive‐native paired observations that fell within it, based on the associated root traits. Red bars indicate invasive plants have a significantly higher trait value compared to native plants; light blue bars denote that invasive plants have a significantly lower trait value than native plants; gray bars indicate mixed results. The numbers shown within the bars provide the number of paired observations supporting that result within a given environmental context.

From the 22 articles that fit our criteria, we identified 59 paired comparisons of invasive and native plant root traits across varying levels of disturbance and nutrient availability (Figure 1). Most research focused on low‐disturbance, high‐nutrient environments (*n* = 33), while the other three scenarios had fewer than 15 paired observations each, with the second highest being high disturbance–low nutrient (*n* = 13), followed by low disturbance–low nutrient (*n* = 7), and high disturbance–high nutrient (*n* = 6). Root biomass was the most studied trait (*n* = 22), with far fewer studies examining specific root length (*n* = 9), root‐to‐shoot ratio (*n* = 8), or root diameter (*n* = 7). Only a few other traits had more than a handful of comparisons, and many were absent across multiple environmental scenarios. We synthesize the patterns within each quadrant below and present the corresponding RTEI predictions derived from the existing literature.

### 4.1 Scenario 1: Low‐Disturbance and High‐Nutrient Availability Environments

Plant invaders in low‐disturbance and high‐nutrient environments exhibited no clear patterns except for root biomass (Figure 1a). All but one paired observation (89%) indicated that invasive plants had significantly higher root biomass than native plants in low‐disturbance and high‐nutrient environments. All other root traits showed similar proportions of positive, negative, and mixed results when comparing invasive and native root traits.

**FIGURE 2:**
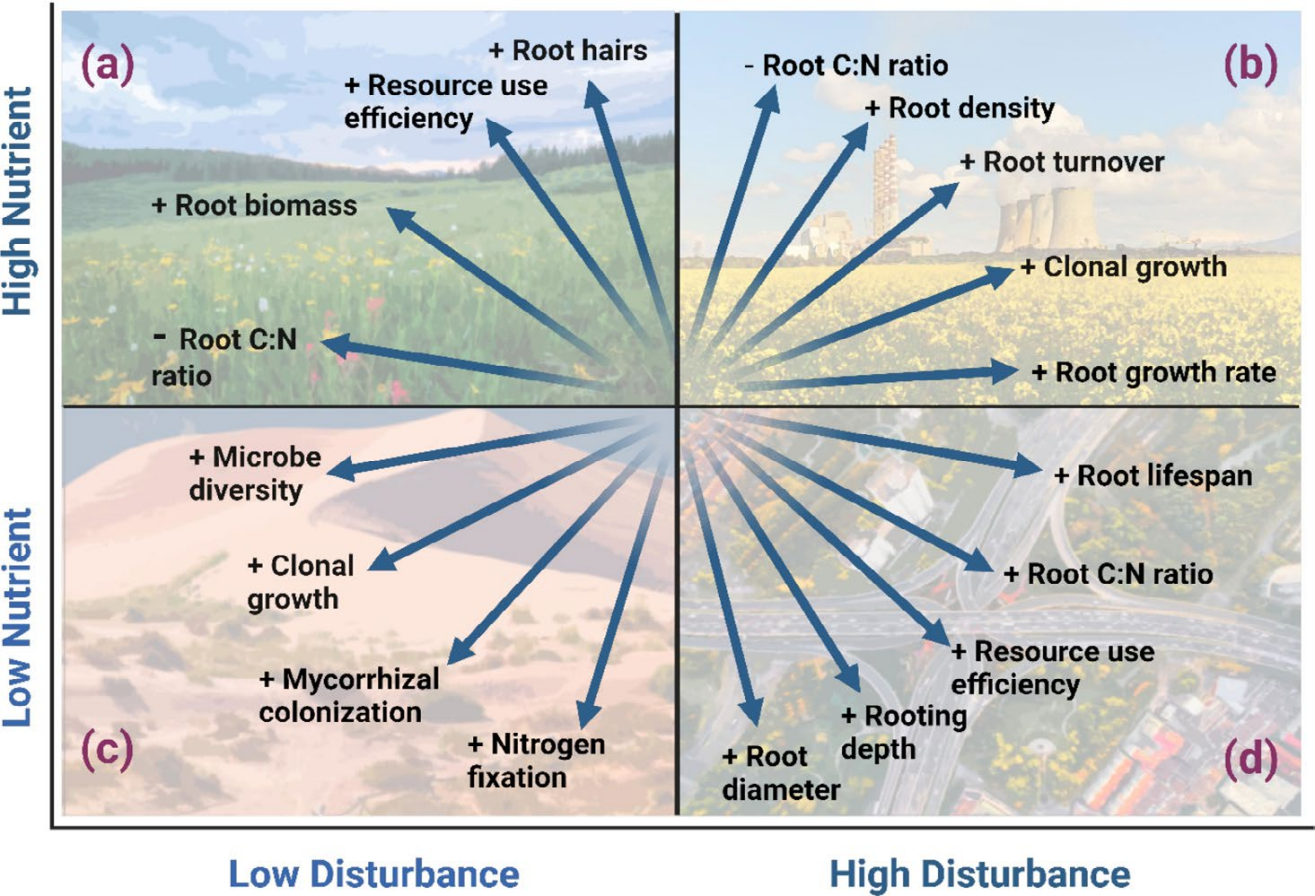
Conceptual diagram illustrating the Root Trait–Environment–Invasion (RTEI) Framework. Hypothesized root traits are represented across four environmental scenarios: (a) low‐disturbance and high‐nutrient availability, (b) high‐disturbance and high‐nutrient availability, (c) low‐disturbance and low‐nutrient availability, and (d) high‐disturbance and low‐nutrient availability. The arrows point to each trait (in bold black text) hypothesized to aid invasive plant performance in a specific ecological context. The “+” symbol indicates a comparative increase in a specific trait that would likely enhance invasive plant outcomes. The “−” symbol indicates a comparative decrease in a specific trait that might increase invasive plant outcomes. Figure credits: BioRender.com.

Although the empirical patterns were largely inconsistent across most root traits in our review, the strong signal for increased root biomass suggests a potential acquisitive strategy that underpins our RTEI framework (Figure 2). In low‐disturbance environments with rich soil nutrients, such as temperate or boreal old‐growth forests, nonnative plant invaders will likely face fierce competition from established, highly competitive native species. In such ecosystems, release from natural enemies can minimize the energy required for defense in invasive plants (Blumenthal 2005), resulting in traits related to fast resource utilization and acquisition (Caplan et al. 2019), such as high root biomass and specific root length (Figure 2a). Increased root hair density could further support exudate production and nutrient uptake by increasing the available root surface area without incurring the high costs associated with maintenance (Holz et al. 2018) (Figure 2a). In high‐nutrient environments, invasive plants can reduce their investment in resource conservation, lowering C:N ratios and increasing root turnover, potentially minimizing root maintenance cost while enhancing resource acquisition (James et al. 2010).

### 4.2 Scenario 2: High‐Disturbance and High‐Nutrient Availability Environments

Only a handful of studies investigated how native and invasive root traits differed under conditions where disturbance and access to nutrients were high (*n* = 6 paired observations; Figure 1b). Of the six paired observations, there were no clear patterns of how invasive and native plants differed in their root traits, with root biomass and specific root length having equal proportions of positive and mixed results (Figure 1b).

Despite the lack of clear empirical patterns, ecological theory and known disturbance–resource dynamics allow us to predict which root traits should confer an advantage in high‐disturbance, high‐nutrient environments. In high‐disturbance and high‐nutrient conditions like fragmented temperate grasslands, our RTEI framework hypothesizes that root traits associated with fast, aggressive, and efficient acquisition of belowground resources will facilitate greater performance from invasives due to divergent root trait strategies (Figure 2b). High‐disturbance environments can change nutrient availability dramatically (Davis et al. 2000); hence, traits related to fast root growth, high root turnover via low C:N ratios, and efficient resource acquisition of belowground resources would enable invasive plants to acquire resources once they become available (Lin et al. 2018). Another belowground trait we consider in high‐disturbance and high‐nutrient environments is root clonal regeneration, which allows for enhanced persistence, rapid reproduction, and spread via stolon or root fragments (Dong et al. 2012) (Figure 2b). Vegetative reproduction has been identified as a major driver of invasive success for some plant invaders (Nunez‐Mir et al. 2019).

### 4.3 Scenario 3: Low‐Disturbance and Low‐Nutrient Availability Environments

As in low‐disturbance, high‐nutrient environments, we found few studies (*n* = 7 paired observations) investigating the effects of invader root traits in ecosystems with low disturbance and nutrient availability. These studies reported mixed results on differences in nitrogen‐use efficiency and root‐to‐shoot ratio between invasive and native species (Figure 1c). Root biomass showed an equal proportion of positive and mixed results.

In light of these limited and mixed findings, we propose mechanistic RTEI hypotheses for how root traits may mediate invasive plant performance in low‐disturbance, low‐nutrient systems. Ecosystems characterized by low disturbance and low nutrient availability, such as those typical of remote Arctic and arid climates, are generally difficult for nonnative plants to invade. Only plants adapted to these harsh conditions can survive, which is why native plants that evolved under these conditions will have a competitive advantage over invading nonnatives (Hiltbrunner et al. 2014). However, plant invasions have been observed in these nutrient‐poor ecosystems, including some of the most remote places on Earth (e.g., Antarctica; Hughes et al. 2020). We hypothesize that root traits that encourage microbial symbioses (e.g., mycorrhizal colonization and N fixation via rhizobia) will facilitate greater performance for invasive plants by increasing soil fertility and biodiversity (Figure 2c). Plant invaders can enhance their nutrient uptake in this scenario with root traits such as N fixation, mycorrhizal colonization, or microbial diversity, thereby increasing their invasiveness. We also hypothesize that clonal reproduction from roots will be an important trait, enabling invasive plants to spread without the high‐energetic cost of sexual reproduction (Figure 2c).

### 4.4 Scenario 4: High‐Disturbance and Low‐Nutrient Availability Environments

Our review yielded a moderate number of paired observations (*n* = 13) in high‐disturbance, low‐nutrient ecosystems. Across three paired observations, invasive plants displayed greater specific root length and root tissue density than native plants (Figure 1d). Apart from root biomass, which showed no clear pattern, no other root traits were represented by more than two paired observations.

Though empirical support remains limited in this scenario, the ecological context of high disturbance under nutrient limitation provides a strong foundation for generating new RTEI hypotheses. High disturbance creates substantial temporal and spatial variation in resource availability and open space (Alpert et al. 2000; Davis et al. 2005), a dynamic that may be especially important in nutrient‐poor ecosystems such as coastal dunes and deserts. Thus, we hypothesize that successful invaders in these ecosystems possess root traits that enable them to take advantage of transient resources while maintaining performance under chronically low nutrient conditions (Figure 2d). Traits consistent with this strategy may include greater root C:N ratios, lifespan, diameter, depth, and resource‐use efficiency, all of which are associated with enhanced persistence and resource conservation under nutrient limitation (Figure 2d). At the same time, disturbance may periodically generate short‐lived resource pulses, creating opportunities for traits associated with resource acquisition, such as high SRL, rapid root growth, and early phenology/root deployment (Lau and Funk 2023). Together, these contrasting selective pressures suggest that invaders in highly disturbed, nutrient‐poor environments may require a combination of traits that balance persistence under resource scarcity with the capacity to rapidly exploit ephemeral resource opportunities.

## 5 Future Research Directions

Our RTEI framework lays theoretical groundwork for linking root trait strategies to invasion outcomes across diverse environmental contexts, yet direct empirical tests remain limited. The paucity of empirical studies examining root trait syndromes across disturbance and resource‐availability scenarios hinders our ability to generalize findings and anticipate future plant invaders. Consequently, the current synthesis was limited to comparing root traits between invasive and native plants across different environmental conditions rather than explicitly testing the RTEI framework in each of its four proposed scenarios. For this reason, our analysis can identify trait syndromes associated with invasiveness, but it cannot fill the current gap in scenario‐specific predictions about which traits confer invasion success. Instead, our results describe divergent root trait syndromes between invasive and native species, without determining whether these traits confer competitive advantages. Furthermore, many root traits remain underrepresented in the literature, with root biomass the only trait sufficiently examined across all four scenarios. Advancing the RTEI framework will require targeted research that directly evaluates how coordinated root trait syndromes mediate invasiveness across gradients of disturbance and nutrient availability.

We first advocate for studies that examine whether invasion success reflects inherently superior trait values or context‐dependent trait–environment matching. Explicit tests of our RTEI framework require factorial experiments manipulating disturbance regimes and nutrient availability while introducing species with well‐characterized root economic strategies. Second, we advocate comparing aboveground and belowground traits across these two axes in these experiments. Such designs would fill in the empirical knowledge gap and enable researchers to determine whether invasion success reflects trait superiority, phenotypic plasticity, environmental filtering, or their interaction.

Future research must also account for covariance, trade‐offs, and potential collinearity among root traits, as well as between belowground and aboveground traits. Plant traits rarely operate independently; instead, they are often coordinated along the economic spectrum or across multidimensional strategy axes (e.g., acquisitive vs. conservative investment [Roumet et al. 2016], structural allocation vs. symbiotic reliance [Bergmann et al. 2020]). Trade‐offs may constrain viable trait combinations, thereby shaping which invasion strategies are ecologically feasible. Moreover, collinearity among traits can obscure statistical inference and inflate predictive models if not adequately addressed. Multivariate approaches—such as principal component analyses, structural equation modeling, trait network analyses, and covariance‐based modeling—are therefore essential for testing whether invasion success reflects specific traits or integrated trait syndromes.

Lastly, the field lacks clarity on whether root traits are causes or consequences of invasion. Most studies measure traits after establishment, making directionality difficult to infer. Introducing nonnative species with characterized root traits into intact native communities, alongside noninvasive congeners, would help disentangle pre‐adaptation from post‐invasion plastic responses. Embedding these comparisons within a phylogenetic framework can further test whether invasion success reflects evolutionary distinctiveness or functional convergence (sensu Darwin's naturalization hypothesis).

By addressing these conceptual and methodological challenges, such as trait trade‐offs, multivariate trait structure, and environmental contingency, future research can substantially improve the predictive power and ecological utility of the RTEI framework.

## Author Contributions

**Amoi Campbell:** conceptualization (lead), data curation (lead), methodology (lead), writing – original draft (lead), writing – review and editing (lead). **Jennifer L. Funk:** conceptualization (supporting), visualization (supporting), writing – original draft (supporting), writing – review and editing (supporting). **Sara Kuebbing:** conceptualization (supporting), writing – original draft (supporting), writing – review and editing (supporting). **Martin A. Nuñez:** conceptualization (supporting), writing – original draft (supporting), writing – review and editing (supporting). **Gabriela C. Nunez‐Mir:** conceptualization (supporting), writing – original draft (supporting), writing – review and editing (supporting). **Matthew A. McCary:** conceptualization (supporting), data curation (supporting), methodology (supporting), visualization (supporting), writing – original draft (supporting), writing – review and editing (supporting).

## Funding

This work was supported by the NSF Division of Environmental Biology (2440649).

## Conflicts of Interest

The authors declare no conflicts of interest.

## Supporting information


**Appendix S1:** The full list of articles included/excluded based on our inclusion criteria. Table S1 = List of eligible articles; Table S2 = Articles screened after 1 round; Table S3 = Articles screened after full‐text evaluation.

**Appendix S2:** The full search methods and overview of the systematic literature review supporting the main text.

## Data Availability

All data and R code supporting this study are publicly available on Zenodo at https://doi.org/10.5281/zenodo.21480538.
