# Supplementary material for: From the Ground Up: Can Root Traits Help Understand Invasion Dynamics?
Source: Ecol Evol. 2026 Jul 31;16(8):e74107. doi: 10.1002/ece3.74107 (PMC13426332; doi:10.1002/ece3.74107)
Supplement: Supplementary file 1 — Appendix S1: The full list of articles included/excluded based on our inclusion criteria. Table S1 = List of eligible articles; Table S2 = Articles screened after 1 round; Table S3 = Articles screened after full‐text evaluation. [file ECE3-16-e74107-s001.docx]

**Appendix S1.** The full list of articles included/excluded based on our inclusion criteria. Table S1 = List of eligible articles (pages 1-4); Table S2 = Articles screened after 1 round (pages 5-24); Table S3 = Articles screened after full-text evaluation (pages 25-38)

**Table S1.** List of eligible studies used in the vote-count meta-analysis. The “Disturbance” and “Nutrient” columns refer to the scenarios that were observed in the respective study.

| **Study** | **Authors** | **Year** | **Journal** | **Source** | **Location** | **Ecosystem** | **Study type** | **Invasive Species** | **Disturbance** | **Nutrient** |
| --- | --- | --- | --- | --- | --- | --- | --- | --- | --- | --- |
| 1 | Broadbent et al. DOI: https://doi.org/10.1007/s00442-017-4039-5 | 2018 | *OECOLOGIA* | Google  Scholar | Canterbury, New Zealand | Grassland | Greenhouse | *Agrostis capillaris* | none | nitrogen addition |
| 2 | Caplan et al. DOI:  https://doi.org/10.1002/ecy.2588 | 2019 | *ECOLOGY* | Google  Scholar | New Jersey, USA | Old-field grassland/  successional forest | Observational field study | Species mix | none | high resources: abandoned agricultural field |
| 3 | Caplan & Yeakley  DOI: https://doi.org/10.1007/s00442-013-2639-2 | 2013 | *OECOLOGIA* | Google  Scholar | Oregon, USA | Grassland | Greenhouse | Species mix | drought | fertilized soil |
| 4 | Leonard et al.  DOI: doi:10.1614/IPSM-07-038.1 | 2008 | *INVASIVE PLANT SCIENCE AND MANAGEMENT* | Web of  Science | Utah, USA | Old-field grassland | Field experiment | *Bromus tectorum* | none | high resources: abandoned agricultural field |
| 5 | Barros et al.  DOI: https://doi.org/10.1016/j.plaphy.2019.12.018 | 2020 | *PLANT PHYSIOLOGY AND BIOCHEMISTRY* | Google  Scholar | Northeastern Brazil | Dry Tropical Forest | Greenhouse | *Prosopis juliflora* | drought | none |
| 6 | Li et al.  DOI: https://doi.org/10.1007/s11104-022-05734-5 | 2022 | *PLANT AND SOIL* | Web of  Science | Kaifeng, China | Grassland | Field experiment | Species mix | drought | none |
| 7 | Osone et al.  DOI: https://doi.org/10.1007/s10265-013-0609-8 | 2014 | *JOURNAL OF PLANT RESEARCH* | Web of  Science | Bonin Islands, Japan | Subtropical  forest | Greenhouse | *Bichofia javanica* | none | nitrogen addition |
| 8 | Kolodziejek  DOI: https://doi.org/10.1038/s41598-019-39947-z | 2019 | *SCIENTIFIC REPORTS* | Web of  Science | Lodz, Poland | Grassland | Field experiment | *Rumex confertus* | none | fertilized soil |
| 9 | Ali & Bucher  DOI: https://doi.org/10.1002/ece3.9296 | 2022 | *ECOLOGY AND EVOLUTION* | Web of  Science | Lake Manzala, Egypt | Mediterranean grassland | Field experiment | *Imperata cylindrica* | drought | none |
| 10 | Blicker et al.  DOI: https://doi.org/10.1023/A:1021596627967 | 2002 | *PLANT AND SOIL* | Google  Scholar | Montana, USA | Semi-arid grasslands | Greenhouse | *Centaurea maculosa* | none | low N addition |
| 11 | Matzek  DOI: https://doi.org/10.1007/s10530-011-9985-y | 2011 | *BIOLOGICAL INVASIONS* | Google  Scholar | California, USA | Forests | Greenhouse | Species mix | none | resource-limited habitat |
| 12 | Bueno et al.  DOI: https://doi.org/10.1093/treephys/tpaa134 | 2021 | *TREE PHYSIOLOGY* | Web of  Science | Konstanz, Germany | Temperate  forest | Greenhouse | Species mix | drought | none |
| 13 | Radford et al.  DOI: https://doi.org/10.1111/j.1442-9993.2007.01700.x | 2007 | *AUSTRAL ECOLOGY* | Google  Scholar | California, USA | Montane  grassland | Greenhouse | Species mix | none | nitrogen addition |
| 14 | Valliere et al.  DOI: https://doi.org/10.1111/1365-2435.14161 | 2022 | *FUNCTIONAL ECOLOGY* | Web of  Science | California, USA | Coastal sage  scrub | Greenhouse | Species mix | none | low nutrients |
| 16 | DeFalco et al.  DOI: https://doi.org/10.3732/ajb.90.7.1045 | 2003 | *AMERICAN JOURNAL OF BOTANY* | Google  Scholar | Nevada, USA | Desert  grassland | Greenhouse | *Bromus rubens* | none | nitrogen addition |
| 17 | Han et al.  https://doi.org/10.1007/s11258-012-0070-0 | 2012 | *American Journal of Botany* | Google  Scholar | Brisbane, Australia | Grassland | Greenhouse | *Eragrostis curvula* | none | low nutrients |
| 18 | Miao & Bazzaz  DOI: https://doi.org/10.2307/1938630 | 1990 | *Ecology* | Google  Scholar | Massachusetts, USA | Grassland | Greenhouse | *Plantago major* | none | fertilized soil |
| 19 | Leishman and Thomson  DOI: https://doi.org/10.1111/j.1365-2745.2004.00938.x | 2005 | *Journal of Ecology* | Google  Scholar | Sydney, Australia | Woodland | Greenhouse | Species mix | none | nutrient addition |
| 20 | Witkowski  DOI: https://www.jstor.org/stable/2389560 | 1991 | *British Ecological Society* | Google  Scholar | Cape Town, South Africa | Coastal  fynbos | Greenhouse | *Acacia saligna* | none | nutrient addition |
| 21 | Drenovsky et al.  DOI: https://doi.org/10.3732/ajb.1100417 | 2012 | *American Journal of Botany* | Google  Scholar | Intermountain West of the USA | Grassland | Greenhouse | *Centaurea stoebe;* *Linaria dalmatica* | drought | nitrogen addition |
| 22 | Funk  DOI: https://doi.org/10.1111/j.1365-2745.2008.01435.x | 2008 | *Journal of Ecology* | Google  Scholar | Hawaii, USA | Tropical  forest | Greenhouse | Species mix | limited access to light | nitrogen addition |

**Table S2.** First round of screening articles using title names and associated abstracts.

| **Authors** | **Year** | **Title** | **Journal** | **Source** |
| --- | --- | --- | --- | --- |
| Garbowski et al. | 2020 | Getting to the root of restoration: considering root traits for improved restoration outcomes under drought and competition | RESTORATION ECOLOGY | Web of Science |
| James | 2008 | Effect of soil nitrogen stress on the relative growth rate of annual and perennial grasses in the Intermountain West | PLANT AND SOIL | Web of Science |
| Pfadenhauer et al. | 2023 | Remember your roots: Biogeographic properties of plants' native habitats can inform invasive plant risk assessments | DIVERSITY AND DISTRIBUTIONS | Web of Science |
| Huang et al. | 2017 | Performance of the invasive Eupatorium catarium and Ageratum conyzoides in comparison with a common native plant under varying levels of light and moisture | WEED BIOLOGY AND MANAGEMENT | Web of Science |
| MacDougall & Turkington | 2005 | Are invasive species the drivers or passengers of change in degraded ecosystems? | ECOLOGY | Web of Science |
| Ammondt & Litton | 2012 | Competition between Native Hawaiian Plants and the Invasive Grass Megathyrsus maximus: Implications of Functional Diversity for Ecological Restoration | RESTORATION ECOLOGY | Web of Science |
| Bui et al. | 2016 | Phragmites australis: How do genotypes of different phylogeographic origins differ from their invasive genotypes in growth, nitrogen allocation and gas exchange? | BIOLOGICAL INVASIONS | Web of Science |
| Jeudy et al. | 2016 | RhizoTubes as a new tool for high throughput imaging of plant root development and architecture: test, comparison with pot grown plants and validation | PLANT METHODS | Web of Science |
| Zhang et al. | 2022 | Differences in Phenotypic Plasticity between Invasive and Native Plants Responding to Three Environmental Factors | LIFE-BASEL | Web of Science |
| Ilyas et al. | 2022 | Leaf Functional Traits of Invasive Grasses Conferring High-Cadmium Adaptation Over Natives | FRONTIERS IN PLANT SCIENCE | Web of Science |
| van Kleunen et al. | 2011 | Preadapted for invasiveness: do species traits or their plastic response to shading differ between invasive and non-invasive plant species in their native range? | JOURNAL OF BIOGEOGRAPHY | Web of Science |
| Luong & Loik | 2022 | Adjustments in physiological and morphological traits suggest drought-induced competitive release of some California plants | ECOLOGY AND EVOLUTION | Web of Science |
| Barros et al. | 2021 | Low foliar construction cost and strong investment in root biomass in Calotropis procera, an invasive species under drought and recovery | FLORA | Web of Science |
| Gioia et al. | 2017 | GrowScreen-PaGe, a non-invasive, high-throughput phenotyping system based on germination paper to quantify crop phenotypic diversity and plasticity of root traits under varying nutrient supply | FUNCTIONAL PLANT BIOLOGY | Web of Science |
| Ulloa et al. | 2016 | Analysis of root-knot nematode and fusarium wilt disease resistance in cotton (Gossypium spp.) using chromosome substitution lines from two alien species | GENETICA | Web of Science |
| Leger & Goergen | 2017 | Invasive Bromus tectorum alters natural selection in arid systems | JOURNAL OF ECOLOGY | Web of Science |
| Ehrenfeld, JG | 2004 | Implications of invasive species for belowground community and nutrient | WEED TECHNOLOGY | Web of Science |
| Hovick et al. | 2011 | Purple loosestrife suppresses plant species colonization far more than broad-leaved cattail: experimental evidence with plant community implications | JOURNAL OF ECOLOGY | Web of Science |
| Harvey & Leffler | 2020 | Differential stoichiometric homeostasis and growth in two native and two invasive C(3)grasses | OECOLOGIA | Web of Science |
| Wang et al. | 2017 | Effects of light intensity on morphology and physiology of exotic invasive Bidens pilosa L. and non-invasive congener Bidens bipinnata L. | ALLELOPATHY JOURNAL | Web of Science |
| Lombardi et al. | 2021 | Ground-Penetrating Radar as phenotyping tool for characterizing intraspecific variability in root traits of a widespread conifer | PLANT AND SOIL | Web of Science |
| Baruch et al. | 2000 | Responses to light and water availability of four invasive Melastomataceae in the Hawaiian islands | INTERNATIONAL JOURNAL OF PLANT SCIENCES | Web of Science |
| James | 2008 | Effect of soil nitrogen stress on the relative growth rate of annual and perennial grasses in the Intermountain West | PLANT AND SOIL | Web of Science |
| Nagel et al. | 2012 | GROWSCREEN-Rhizo is a novel phenotyping robot enabling simultaneous measurements of root and shoot growth for plants grown in soil-filled rhizotrons | FUNCTIONAL PLANT BIOLOGY | Web of Science |
| Armengaud et al. | 2009 | EZ-Rhizo: integrated software for the fast and accurate measurement of root system architecture | PLANT JOURNAL | Web of Science |
| Sun et al. | 2013 | Non-invasive microelectrode cadmium flux measurements reveal the spatial characteristics and real-time kinetics of cadmium transport in hyperaccumulator and nonhyperaccumulator ecotypes of Sedum alfredii | JOURNAL OF PLANT PHYSIOLOGY | Web of Science |
| Jerszurk et al. | 2017 | Impact of root growth and hydraulic conductance on canopy carbon-water relations of young walnut trees (Juglans regia L.) under drought | SCIENTIA HORTICULTURAE | Web of Science |
| Orman-Ligeza et al. | 2018 | The Xerobranching Response Represses Lateral Root Formation When Roots Are Not in Contact with Water | CURRENT BIOLOGY | Web of Science |
| Koziol et al. | 2012 | REDUCED DROUGHT TOLERANCE DURING DOMESTICATION AND THE EVOLUTION OF WEEDINESS RESULTS FROM TOLERANCE-GROWTH TRADE-OFFS | EVOLUTION | Web of Science |
| Liu et al. | 2015 | Phenotypic effects of additional chromosomes on agronomic and photosynthetic traits of common wheat in the background of Chinese Spring | CROP & PASTURE SCIENCE | Web of Science |
| Awasthi | 2018 | Morph-physiological responses of cotton interspecific chromosome substitution lines to low temperature and drought stresses | EUPHYTICA | Web of Science |
| Leffler et al. | 2013 | Temperature and functional traits influence differences in nitrogen uptake capacity between native and invasive grasses | OECOLOGIA | Web of Science |
| Boughton et al. | 2016 | Reproductive traits of Lachnanthes caroliniana (Lam.) Dandy related to patch formation following feral swine rooting disturbance | JOURNAL OF THE TORREY BOTANICAL SOCIETY | Web of Science |
| Huang et al. | 2022 | Functional traits underlying performance variations in the overwintering of the cosmopolitan invasive plant water hyacinth (Eichhornia crassipes) under climate warming and water drawdown | ECOLOGY AND EVOLUTION | Web of Science |
| Kimball et al. | 2016 | Can functional traits predict plant community response to global change? | ECOSPHERE | Web of Science |
| Yu, Hailun et al. | 2021 | Drought Stress Influences the Growth and Physiological Characteristics of Solanum rostratum Dunal Seedlings From Different Geographical Populations in China | FRONTIERS IN PLANT SCIENCE | Web of Science |
| Korres et al. | 2016 | Cultivars to face climate change effects on crops and weeds: a review | AGRONOMY FOR SUSTAINABLE DEVELOPMENT | Web of Science |
| Meyer et al. | 2016 | Strong genetic differentiation in the invasive annual grass Bromus tectorum across the Mojave-Great Basin ecological transition zone | BIOLOGICAL INVASIONS | Web of Science |
| Du et al. | 2019 | Drought affects the coordination of belowground and aboveground resource-related traits in Solidago canadensis in China | ECOLOGY AND EVOLUTION | Web of Science |
| Britez et al. | 2020 | Coppicing of two native but invasive oak species in Florida | FOREST ECOLOGY AND MANAGEMENT | Web of Science |
| Rowe & Leger | 2011 | Competitive seedlings and inherited traits: a test of rapid evolution of Elymus multisetus (big squirreltail) in response to cheatgrass invasion | EVOLUTIONARY APPLICATIONS | Web of Science |
| Kinmonth-Schultz & Kim | 2011 | Carbon gain, allocation and storage in rhizomes in response to elevated atmospheric carbon dioxide and nutrient supply in a perennial C-3 grass, Phalaris arundinacea | FUNCTIONAL PLANT BIOLOGY | Web of Science |
| Krushelnycky et al. | 2020 | Clinal variation in drought resistance shapes past population declines and future management of a threatened plant | ECOLOGICAL MONOGRAPHS | Web of Science |
| Stewart & Graves | 2006 | Photosynthesis, growth, carbon allocation, and fruit load of Frangula caroliniana and Rhamnus cathartica | INTERNATIONAL JOURNAL OF PLANT SCIENCES | Web of Science |
| Leger & Baughman | 2015 | What Seeds to Plant in the Great Basin? Comparing Traits Prioritized in Native Plant Cultivars and Releases with Those That Promote Survival in the Field | NATURAL AREAS JOURNAL | Web of Science |
| Tortorelli et al. | 2022 | Community invasion resistance is influenced by interactions between plant traits and site productivity | ECOLOGY | Web of Science |
| Pratap et al. | 2019 | Using Plant Phenomics to Exploit the Gains of Genomics | AGRONOMY-BASEL | Web of Science |
| Kui et al. | 2019 | Can environmental flows moderate riparian invasions? The influence of seedling morphology and density on scour losses in experimental floods | FRESHWATER BIOLOGY | Web of Science |
| Khan et al. | 2019 | Exploring the Potential of Overexpressed OsCIPK2 Rice as a Nitrogen Utilization Efficient Crop and Analysis of Its Associated Rhizo-Compartmental Microbial Communities | INTERNATIONAL JOURNAL OF MOLECULAR SCIENCES | Web of Science |
| Closset-Kopp et al. | 2007 | When Oskar meets Alice: Does a lack of trade-off in r/K-strategies make Prunus serotina a successful invader of European forests? | FOREST ECOLOGY AND MANAGEMENT | Web of Science |
| Wang et al. | 2016 | Genetic and Epigenetic Alterations of Brassica nigra Introgression Lines from Somatic Hybridization: A Resource for Cauliflower Improvement | FRONTIERS IN PLANT SCIENCE | Web of Science |
| Timmusk et al. | 2014 | Drought-Tolerance of Wheat Improved by Rhizosphere Bacteria from Harsh Environments: Enhanced Biomass Production and Reduced Emissions of Stress Volatiles | PLOS ONE | Web of Science |
| Bai et al. | 2021 | Identification of genetic locus with resistance to take-all in the wheat-Psathyrostachys huashanica Keng introgression line H148 | JOURNAL OF INTEGRATIVE AGRICULTURE | Web of Science |
| Fioran et al. | 2013 | Future Scenarios for Plant Phenotyping | ANNUAL REVIEW OF PLANT BIOLOGY, VOL 64 | Web of Science |
| Foulkes et al. | 2011 | Raising yield potential of wheat. III. Optimizing partitioning to grain while maintaining lodging resistance | JOURNAL OF EXPERIMENTAL BOTANY | Web of Science |
| Mueller et al. | 2017 | Ecosystem consequences of plant genetic divergence with colonization of new habitat | ECOSPHERE | Web of Science |
| Zhang et al. | 2021 | Scaling up experimental stress responses of grass invasion to predictions of continental-level range suitability | ECOLOGY | Web of Science |
| Gao et al. | 2022 | Comparative differences in photosynthetic characteristics, ion balance, and nitrogen metabolism between young and old wild soybean leaves under nitrogen deficiency | PLANT STRESS | Web of Science |
| He et al. | 2014 | A systems-wide comparison of red rice (Oryza longistaminata) tissues identifies rhizome specific genes and proteins that are targets for cultivated rice improvement | BMC PLANT BIOLOGY | Web of Science |
| Malik et al. | 2020 | Cassava breeding and agronomy in Asia: 50 years of history and future directions | BREEDING SCIENCE | Web of Science |
| Prior et al. | 2017 | When does invasive species removal lead to ecological recovery? Implications for management success | Biological Invasions | Google Scholar |
| Pickett & Cadenasso | 2008 | Altered resources, disturbance, and heterogeneity: A framework for comparing urban and non-urban soils | Urban Ecosystems | Google Scholar |
| Haddad et al. | 2015 | Habitat fragmentation and its lasting impact on Earth’s ecosystems | Science Advances | Google Scholar |
| Funk & Vitousek | 2007 | Resource-use efficiency and plant invasion in low-resource systems | Nature | Google Scholar |
| White & Pickett | 1985 | The Ecology of Natural Disturbance and Patch Dynamics | Academic Press | Google Scholar |
| Alpert et al. | 2000 | Invasiveness, invasibility and the role of environmental stress in the spread of non-native plants | Perspectives in Plant Ecology, Evolution and Systematics | Google Scholar |
| Haddad et al. | 2015 | Habitat fragmentation and its lasting impact on Earth’s ecosystems | Science Advances | Google Scholar |
| Ehrenfeld | 2003 | Effects of exotic plant invasions on soil nutrient cycling processes | Ecosystems | Google Scholar |
| Grimm et al | 2016 | Does the ecological concept of disturbance have utility in urban social–ecological–technological systems? | Ecosystem Health and Sustainability | Google Scholar |
| Richardson et al | 2001 | Naturalization and invasion of alien plants: Concepts and definitions | Diversity Distributions | Google Scholar |
| Borden & Flory | 2021 | Urban evolution of invasive species | Frontiers in Ecology and the Environment | Google Scholar |
| Davis et al. | 2000 | Fluctuating resources in plant communities: a general theory of invasibility | Journal of Ecology | Google Scholar |
| Nicotra and Davidson | 2010 | Adaptive phenotypic plasticity and plant water use | Functional Plant Biology | Google Scholar |
| Wisley & Polley | 2006 | Aboveground productivity and root–shoot allocation differ between native and introduced grass species | Oecologia | Google Scholar |
| Gioria & Ousborne | 2014 | Resource competition in plant invasions: emerging patterns and research needs | Frontiers in Plant Scienecs | Google Scholar |
| `Miller et al | 2021 | How disturbance history alters invasion success: biotic legacies and regime change | Ecology Letters | Google Scholar |
| Johnstone et al | 2016 | Changing disturbance regimes, ecological memory, and forest resilience | Frontiers in Ecology and the Environment | Google Scholar |
| Orban et al | 2021 | The role of disturbance in invasive plant establishment in a changing climate: insights from a drought experiment | Biological Invasions | Google Scholar |
| Gross et al | 2005 | Grassland Invasibility and Diversity: Responses to Nutrients, Seed Input, and Disturbance | Ecology | Google Scholar |
| Gibbons et al. |  | Invasive Plants Rapidly Reshape Soil Properties in a Grassland Ecosystem | mSystems | Google Scholar |
| Gornish et al | 2020 | Buffelgrass invasion and glyphosate effects on desert soil microbiome communities | Biological Invasions | Google Scholar |
| Heberling & Fridley | 2016 | Invaders do not require high resource levels to maintain physiological advantages in a temperate deciduous forest | New Phytologist | Google Scholar |
| Montesinos | 2022 | Fast invasives fastly become faster: Invasive plants align largely with the fast side of the plant economics spectrum | Journal of Ecology | Google Scholar |
| Brooks | 2003 | Effects of increased soil nitrogen on the dominance of alien annual plants in the Mojave Desert | Journal of Applied Ecology | Google Scholar |
| Richardson & Pyšek | 2012 | Naturalization of introduced plants: ecological drivers of biogeographical patterns | New Phytologist | Google Scholar |
| Daehler | 2003 | Performance Comparisons of Co-Occurring Native and Alien Invasive Plants: Implications for Conservation and Restoration | Annual Reviews | Google Scholar |
| Kueffer et al. | 2013 | Integrative invasion science: model systems, multi-site studies, focused meta-analysis and invasion syndromes | New Phytologist | Google Scholar |
| Blumenthal | 2005 | Interrelated Causes of Plant Invasion | Science | Google Scholar |
| Davis | 2009 | Invasion Biology | Oxford University Press on Demand | Google Scholar |
| Marshall et al | 2012 | Buffel grass (Cenchrus ciliaris) as an invader and threat to biodiversity in arid environments: A review. | Journal of Arid Environments | Google Scholar |
| Ibáñez et al | 2021 | Assessing vulnerability and resistance to plant invasions: a native community perspective | Invasive Plant Science and Management | Google Scholar |
| Pringle et al | 2009 | Mycorrhizal Symbioses and Plant Invasions. | Annual Review of Ecology, Evolution, and Systematics | Google Scholar |
| Shea et al | 2002 | Community ecology theory as a framework for biological invasions | Trends in Ecology & Evolution | Google Scholar |
| Vilà et al | 2011 | Ecological impacts of invasive alien plants: a meta-analysis of their effects on species, communities and ecosystems | Ecology of Letters | Google Scholar |
| Crystal-Ornelas & Lockwood | 2020 | Cumulative meta-analysis decline effect evidence synthesis invasive species species richness stability sufficiency | Ecology | Google Scholar |
| Brousseau et al | 2018 | On the development of a predictive functional trait approach for studying terrestrial arthropods | Journal of Animal Ecology | Google Scholar |
| Violle et al | 2007 | Let the concept of trait be functional! | Oikos | Google Scholar |
| Rejmanek et al | 2005 | Ecology of invasive plants: state of the art | Invasive Alien Species: a New Synthesis | Google Scholar |
| Kourtev et al. | 2002 | Exotic Plant Species Alter the Microbial Community Structure and Function in the Soil | Ecology | Google Scholar |
| Meinhardt and Gehring | 2012 | Disrupting mycorrhizal mutualisms: A potential mechanism by which exotic tamarisk outcompetes native cottonwoods | Ecological Applications | Google Scholar |
| Hulme | 2009 | Trade, transport and trouble: managing invasive species pathways in an era of globalization | Journal of Applied Ecology | Google Scholar |
| Warren et al | 2011 | The interaction between propagule pressure, habitat suitability and density-dependent reproduction in species invasion | Oikos | Google Scholar |
| Pennings & Callaway | 2000 | THE ADVANTAGES OF CLONAL INTEGRATION UNDER DIFFERENT ECOLOGICAL CONDITIONS: A COMMUNITY-WIDE TEST | Ecology | Google Scholar |
| Peltzer | 2002 | Does clonal integration improve competitive ability? A test using aspen (Populus tremuloides [Salicaceae]) invasion into prairie | AMERICAN JOURNAL OF BOTANY | Google Scholar |
| Estrada et al | 2020 | Clonal integration enhances performance of an invasive grass. | Oikos | Google Scholar |
| Tillman | 1985 | The Resource-Ratio Hypothesis of Plant Succession | The American Society of Naturalists | Google Scholar |
| Tharayil | 2009 | Dual purpose secondary compounds: phytotoxin of Centaurea diffusa also facilitates nutrient uptake | New Phytologist | Google Scholar |
| Chen et al | 2019 | Enrichment of soil rare bacteria in root by an invasive plant Ageratina adenophora | Science of The Total Environment | Google Scholar |
| Hernández et al | 2019 | The effects of nitrogen deposition and invasion on litter fuel quality and decomposition in a Stipa pulchra grassland | Journal of Arid Environments | Google Scholar |
| Callaway & Aschehoug | 2000 | Invasive Plants Versus Their New and Old Neighbors: A Mechanism for Exotic Invasion | Science | Google Scholar |
| Callaway et al | 2008 | Novel weapons: invasive plant suppresses fungal mutualists in America but not in its native Europe | Ecology | Google Scholar |
| Thorpe et al | 2009 | Root Exudate Is Allelopathic in Invaded Community but Not in Native Community: Field Evidence for the Novel Weapons Hypothesis | Journal of Ecology | Google Scholar |
| Catford et al | 2009 | Reducing redundancy in invasion ecology by integrating hypotheses into a single theoretical framework. Diversity and Distributions | Diversity and Distributions | Google Scholar |
| Chakraborty & Li | 2010 | Departure from naturalized to invasive stage: a disturbance-induced mechanism and associated interacting factors | Journal of Plant Ecology | Google Scholar |
| Fattorini & Halle | 2004 | The dynamic environmental filter model: how do filtering effects change in assembling communities after disturbance. | Island Press | Google Scholar |
| Paretti et al | 2021 | Occurrence, fate, and transport of aerially applied herbicides to control invasive buffelgrass within Saguaro National Park Rincon Mountain District, Arizona | US Geological Survey | Google Scholar |
| Hobbs & Huenneke | 1992 | Disturbance, Diversity, and Invasion: Implications for Conservation | Conservation Biology | Google Scholar |
| Ravenek et al. | 2016 | Linking root traits and competitive success in grassland species | Plant and Soil | Google Scholar |
| Keser et al, | 2019 | Invasive clonal plant species have a greater root-foraging plasticity than non-invasive ones | Oecologia | Google Scholar |
| Lie et al | 2016 | Ecological Consequences of Clonal Integration in Plants | Frontiers in plant science | Google Scholar |
| Dawson | 2015 | Release from belowground enemies and shifts in root traits as interrelated drivers of alien plant invasion success: a hypothesis | Ecology and Evolution | Google Scholar |
| Faillace et al. | 2018 | Beneath it all: Size, not origin, predicts belowground comeptitive ability in exotic and native shrubs | Journal of the Torrey Botanical Society | Google Scholar |
| Phillips & Leger | 2015 | Plastic responses of native plant root systems to the presence of an invasive annual grass | American Journal of Botany | Google Scholar |
| Lombardi et al. | 2021 | Ground-Penetrating radar as phenotyping tool for characterizing intraspecific variability in root traits of a widespread conifer | Plant and Soil | Google Scholar |
| Wasaya et al. | 2018 | Root phenotyping for drought tolerance: A review | Argonomy-Basel | Google Scholar |
| Peltzer et al. | 2009 | Punching above their weight: low-biomass non-native species alter soil properties during primary succession | Oikos | Google Scholar |
| Luong & Loik | 2022 | Adjustments in physiological and morphological trait suggest drought-induced competitive release of some California plants | Ecology and Evolution | Google Scholar |
| Sardans et al. | 2017 | Plant invasion is associated with higher plant–soil nutrient concentrations in nutrient-poor environments | Global Change Biology | Google Scholar |
| Caldeira et al. | 2015 | Synergy of extreme drought and shrub invasion reduce ecosystem functioning and resilience in water-limited climates | Scientific Reports | Google Scholar |
| Moles et al | 2007 | A new framework for predicting invasive plant species | Journal of Ecology | Google Scholar |
| Sher & Hyatt | 1999 | The Disturbed Resource-Flux Invasion Matrix: A New Framework for Patterns of Plant Invasion | Biological Invasions | Google Scholar |
| Wright et al | 2015 | Flooding disturbances increase resource availability and productivity but reduce stability in diverse plant communities | Nature Communications | Google Scholar |
| Lynch | 1995 | Root Architecture and Plant Productivity | Plant Physiology | Google Scholar |
| Moles et al | 1999 | A new framework for predicting invasive plant species | Journal of Ecology | Google Scholar |
| Sher & Hyatt | 1997 | The Disturbed Resource-Flux Invasion Matrix: A New Framework for Patterns of Plant Invasion | Biological Invasions | Google Scholar |
| Wright et al | 1995 | Flooding disturbances increase resource availability and productivity but reduce stability in diverse plant communities | Nature Communications | Google Scholar |
| Lynch | 1993 | Root Architecture and Plant Productivity | Plant Physiology | Google Scholar |
| Moles et al | 1991 | A new framework for predicting invasive plant species | Journal of Ecology | Google Scholar |
| Sher & Hyatt | 1989 | The Disturbed Resource-Flux Invasion Matrix: A New Framework for Patterns of Plant Invasion | Biological Invasions | Google Scholar |
| Wright et al | 1987 | Flooding disturbances increase resource availability and productivity but reduce stability in diverse plant communities | Nature Communications | Google Scholar |
| Lynch | 1985 | Root Architecture and Plant Productivity | Plant Physiology | Google Scholar |
| Sher & Hyatt | 1999 | The Disturbed Resource-Flux Invasion Matrix: A New Framework for Patterns of Plant Invasion | Biological Invasions | Google Scholar |
| Davis et al. | 2005 | Invasibility: the local mechanism driving community assembly and species diversity | Ecography | Google Scholar |
| Sokol et al. | 2019 | Evidence for the primacy of living root inputs, not root or shoot litter, in forming soil organic carbon | New Phytologist | Google Scholar |
| Rout & Callaway | 2012 | Interactions between exotic invasive plants and soil microbes in the rhizosphere suggest that ‘everything is not everywhere | Annals of Botany | Google Scholar |
| Hayward et al. | 2015 | Ectomycorrhizal fungal communities coinvading with Pinaceae host plants in Argentina: Gringos bajo el bosque | The New phytologist | Google Scholar |
| Delavaux | 2022 | Nitrogen-fixing symbiotic bacteria act as a global filter for plant establishment on islands | Communications Biology | Google Scholar |
| Grace and Zouhar | 2008 | Wildland fire in ecosystems: fire and nonnative invasive plants | U.S. Department of Agriculture, Forest Service, Rocky Mountain Research Station. | Google Scholar |
| You et al. | 2016 | Propagule Pressure, Habitat Conditions and Clonal Integration Influence the Establishment and Growth of an Invasive Clonal Plant, Alternanthera philoxeroides | . Frontiers in Plant Science | Google Scholar |

**Table S3.** Second round of screening articles using title names and associated abstracts.

| Authors | Year | Title | Journal | Source | Reason for exclusion |
| --- | --- | --- | --- | --- | --- |
| James et al. | 2010 | Exploitation of Nutrient-Rich Soil Patches by Invasive Annual and Native Perennial Grasses | INVASIVE PLANT SCIENCE AND MANAGEMENT | Web of Science | Not enough information on disturbance |
| Rashid & Reshi | 2010 | Does carbon addition to soil counteract disturbance-promoted alien plant invasions? | TROPICAL ECOLOGY | Web of Science | No comparison of native species |
| Pfadenhauer et al. | 2023 | Remember your roots: Biogeographic properties of plants' native habitats can inform invasive plant risk assessments | DIVERSITY AND DISTRIBUTIONS | Web of Science | Data not relevant |
| Phillips & Leger | 2015 | PLASTIC RESPONSES OF NATIVE PLANT ROOT SYSTEMS TO THE PRESENCE OF AN INVASIVE ANNUAL GRASS | AMERICAN JOURNAL OF BOTANY | Web of Science | Native root plasticity paper |
| Hiltbrunner et al. | 2014 | Ecological consequences of the expansion of N2‑fixing plants in cold biomes | Oecologia | Google Scholar | Review paper |
| Guerra-Garcia et al. | 2018 | Biomass Allocation, Plantlet Survival, and Chemical Control of the Invasive Chandelier Plant (Kalanchoe delagoensis) (Crassulaceae) | INVASIVE PLANT SCIENCE AND MANAGEMENT | Web of Science | Water treatment experiment and herbicide experiment not factorial |
| Badalament et al. | 2016 | Seedling growth of a native (Ampelodesmos mauritanicus) and an exotic (Pennisetum setaceum) grass | ACTA OECOLOGICA-INTERNATIONAL JOURNAL OF ECOLOGY | Web of Science | No clear indication of disturbance and/or resource treatment |
| Dietz et al. | 2002a | Regeneration growth of the invasive clonal forb Rorippa austriaca (Brassicaceae) in relation to fertilization and interspecific competition | PLANT ECOLOGY | Web of Science | No native plant for comparison |
| James | 2008 | Leaf nitrogen productivity as a mechanism driving the success of invasive annual grasses under low and high nitrogen supply | JOURNAL OF ARID ENVIRONMENTS | Web of science | Not enough information on disturbance |
| Wan et al. | 2018 | Growth responses of Canada goldenrod (Solidago canadensis L.) to increased nitrogen supply correlate with bioavailability of insoluble phosphorus source | ECOLOGICAL RESEARCH | Web of Science | No comparison to native plants under same treatment |
| Musso et al. | 2021 | Effects of water and nutrient availability on morphological, physiological, and biochemical traits of one invasive and one native grass of a Neotropical savanna | ENVIRONMENTAL AND EXPERIMENTAL BOTANY | Google Scholar | Limited root trait measurements |
| Chavana et al. | 2021 | Local adaptation to continuous mowing makes the noxious weed Solanum elaeagnifolium a superweed candidate by improving fitness and defense traits | SCIENTIFIC REPORTS | Web of Science | No native plant for comparison |
| Imada et al. | 2013 | Vertical distribution of fine roots of Tamarix ramosissima in an arid region of southern Nevada | JOURNAL OF ARID ENVIRONMENTS | Google Scholar | No native plant for comparison |
| Grotkopp & Rejmanek | 2007 | High seedling relative growth rate and specific leaf area are traits of invasive species: Phylogenetically independent contrasts of woody angiospernis | AMERICAN JOURNAL OF BOTANY | Web of Science | Not enough information on disturbance |
| Gupta & Narayan | 2012 | Phenotypic plasticity of Chenopodium murale across contrasting habitat conditions in peri-urban areas in Indian dry tropics: Is it indicative of its invasiveness? | PLANT ECOLOGY | Web of Science | No comparison to native plants under same treatments |
| Welles & Funk | 2021 | Patterns of intraspecific trait variation along an aridity gradient suggest both drought escape and drought tolerance strategies in an invasive herb | ANNALS OF BOTANY | Web of Science | Nutrient variability tested but unclear nutrient treatments |
| Zhang et al. | 2015 | Recurrent Water Level Fluctuation Alleviates the Effects of Submergence Stress on the Invasive Riparian Plant Alternanthera philoxeroides | PLOS ONE | Web of Science | No comparison to native plants under same treatments |
| Assaeed et al. | 2020 | Functional Traits Plasticity of the Invasive Herb Argemone ochroleuca Sweet in Different Arid Habitats | PLANTS-BASEL | Web of Science | No comparison to native plants under same treatments |
| DiTommaso et al. | 2021 | Biomass allocation of Vincetoxicum rossicum and V. nigrum in contrasting competitive environments | AMERICAN JOURNAL OF BOTANY | Web of Science | No comparison to native plants under same treatments |
| Rhodes et al. | 2022 | Guinea Grass Establishment in South Texas Is Driven by Disturbance History and Savanna Structure | RANGELAND ECOLOGY & MANAGEMENT | Web of Science | No comparison to native plants |
| Stanisci et al | 2010 | Leaf and root trait variability of alien and native species along Adriatic coastal dunes (Italy) | Plant Biosystems | Google Scholar | The disturbance is nutrient related; confounded treatments |
| Ni et al | 2018 | Fast seedling root growth leads to competitive superiority of invasive plants | Biological Invasions | Google Scholar | Not enough information on nutrient treatment |
| Du et al. | 2019 | Drought affects the coordination of belowground and aboveground resource-related traits in Solidago canadensis in China | ECOLOGY AND EVOLUTION | Web of Science | No comparison to native plants under same treatments |
| Fridley et al. | 2022 | Fast but steady: An integrated leaf-stem-root trait syndrome for woody forest invaders | ECOLOGY LETTERS | Web of Science | Not enough information on disturbance |
| Aoyama et al. | 2022 | Intraspecific variation in native grass seedling plastic trait response to water stress depends on the context of annual grass invasion | RESTORATION ECOLOGY | Web of Science | Native root response study |
| Rutherford & Arche | 2023 | Trait responses of a grassland shrub invader to altered moisture regimes | PLANT AND SOIL | Web of Science | No comparison to native plants under same treatments |
| Dukes et al. | 2011 | Strong response of an invasive plant species (Centaurea solstitialis L.) to global environmental changes | Ecological Application | Google Scholar | No root trait data |
| Liao et al. | 2007 | Altered ecosystem carbon and nitrogen cycles by plant invasion: a meta-analysis | New Phytologist | Google Scholar | Response to invasion; traits not comprehensively measured |
| Liu &Kleunen | 2017 | Responses of common and rare aliens and natives to nutrient availability and fluctuations | Journal of Ecology | Google Scholar | Not enough information on disturbance |
| Jo et al | 2014 | Linking above- and belowground resource use strategies for native and invasive species of temperate deciduous forests | Biological Invasions | Google Scholar | Not enough information on disturbance |
| Martin et al. | 2009 | Invasion by an exotic tree alters above and belowground ecosystem components | Biological Invasions | Google Scholar | Invasion impact study |
| Marshall et al | 2012 | Buffel grass (Cenchrus ciliaris) as an invader and threat to biodiversity in arid environments: A review | Journal of Arid Environments | Google Scholar | Review paper |
| Sokol et al. | 2019 | Evidence for the primacy of living root inputs, not root or shoot litter, in forming soil organic carbon | New Phytologist | Google Scholar | Invasion impact study |
| Luong & Loik | 2022 | Adjustments in physiological and morphological trait suggest drought-induced competitive release of some California plants | Ecology and Evolution | Google Scholar | Invasion competition response study |
| Weston et al. | 2021 | Potential local adaptation in populations of invasive reed canary grass (Phalaris arundinacea) across an urbanization gradient | Ecology and Evolution | Google Scholar | Single invasive characterization across system; no native plant for comparison |
| Gupta & Narayan | 2012 | Phenotypic plasticity of Chenopodium murale across contrasting habitat conditions in peri-urban areas in Indian dry tropics: Is it indicative of its invasiveness? | Plant Ecology | Google Scholar | Single invasive characterization across system; no native plant comparison |
| Blank et al. | 2015 | Suppression of annual Bromus tectorum by perennial Agropyron cristatum: roles of soil nitrogen availability and biological soil space | Aob Plants | Google Scholar | Suppression of invasive plants for restoration and management |
| Imada et al. | 2013 | Vertical Distribution of fine roots of Tamarix ramosissima in an arid region of southern Nevada | Journal of Arid Environments | Google Scholar | Single invasive characterization across system; no native plant comparison |
| Faillace et al. | 2018 | Beneath it all: Size, not origin, predicts belowground comeptitive ability in exotic and native shrubs | Journal of the Torrey Botanical Society | Google Scholar | Disturbance conditions unclear |
| Paretti et al | 2021 | Occurrence, fate, and transport of aerially applied herbicides to control invasive buffelgrass within Saguaro National Park Rincon Mountain District, Arizona | US Geological Survey | Google Scholar | Suppression of invasive plant for restoration and management |
| Estrada et al | 2020 | Clonal integration enhances performance of an invasive grass. | Oikos | Google Scholar | No native plant for comparison |
| Vitousek et al | 1987 | Biological Invasion by Myrica faya Alters Ecosystem Development in Hawaii | Science | Google Scholar | Invasion impact study |
| Miller et al | 2010 | Ecological impacts of buffel grass (Cenchrus ciliaris L.) invasion in central Australia - Does field evidence support a fire-invasion feedback? | The Rangeland Journal | Google Scholar | No native plant for comparison |
| Caplan et al | 2017 | Nutrient foraging strategies are associated with productivity and population growth in forest shrubs | Annals of Botany | Google Scholar | Nutrient treatment unclear |
| Daehler | 2003 | Performance Comparisons of Co-Occurring Native and Alien Invasive Plants: Implications for Conservation and Restoration | Annual Reviews | Google Scholar | Review |
| Gornish et al | 2020 | Buffelgrass invasion and glyphosate effects on desert soil microbiome communities | Biological Invasions | Google Scholar | No comparison of native species/insufficient root data |
| Soti et al. | 2020 | Soil biotic and abiotic conditions negate invasive species performance in native habitat | Ecological Processes volume | Google Scholar | Comparison of single invasive across native and non-native range |
| Soti et al. | 2014 | Mycorrhizal symbiosis and Lygodium microphyllum Invasion in South Florida - A biogeographic comparison | Symbiosis | Google Scholar | Single plant characterization across systems |
| Mazzola et al. | 2010 | Effects of resource availability and propagule supply on native species recruitment in sagebrush ecosystems invaded by Bromus tectorum. | Biological Invasions | Google Scholar | Limited root trait information |
| Steers et al | 2011 | Can Resource-Use Traits Predict Native vs. Exotic Plant Success in Carbon Amended Soils? | Ecological Applications | Google Scholar | Suppression of invasive plant for restoration and management |
| Funk | 2013 | The physiology of invasive plants in low-resource environments | Conservation Physiology | Google Scholar | Review |
| Seabloom et al | 2003 | Invasion, competitive dominance, and resource use by exotic and native California grassland species | Ecological Applications | Google Scholar | Limited root trait information |
| Wang et al. | 2017 | Disturbance, Trait similarities, and Trait advantages facilitate the invasion success of Altnernanthera philoxeroides (Mart.) Griseb | Clean-Soil Air Water | Google Scholar | Disturbance by nutrient; confounded treatments |
| Lin et al. | 2018 | Evolutionary changes in growth, regrowth and carbohydrate storage in an invasive plant | Scientific Reports | Google Scholar | Defoliation representing herbivory |
