# Supplementary material for: From the Ground Up: Can Root Traits Help Understand Invasion Dynamics?
Source: Ecol Evol. 2026 Jul 31;16(8):e74107. doi: 10.1002/ece3.74107 (PMC13426332; doi:10.1002/ece3.74107)
Supplement: Supplementary file 2 — Appendix S2: The full search methods and overview of the systematic literature review supporting the main text. [file ECE3-16-e74107-s002.docx]

**Appendix S2.** The full search methods and overview of the systematic literature review supporting the main text.

**Literature search**

To identify studies for our systematic review investigating the relationship between invasive and native root traits across environmental contexts (i.e., disturbance and nutrient availability), we conducted a literature search in ISI Web of Science in February 2023 using the following search terms, with no restriction on publication year: (invasive plant OR exotic plant* OR nonnative weed* OR noxious weed* OR invasive weed* OR alien plant*) AND (disturb* OR fire* OR burn* OR prescribed burn* OR drought* OR flood* OR abandon* OR agriculture* OR urban* OR city) AND (nutrient availability* OR high resource* OR low resource* OR nitrogen availability* OR phosphorus availability*) AND (root trait* OR belowground trait* OR belowground competition)*.

This search yielded 108 published articles. We identified 117 additional studies by reviewing reference lists and the publication records of researchers specializing in root traits and plant invasions. Each article’s title and abstract were screened for relevance to the meta-analysis (Appendix S1: Tables S1-S3). No unpublished datasets were included in this study. We followed the PRISMA (Preferred Reporting Items for Systematic Reviews and Meta-Analyses) guidelines (Moher et al., 2009) to conduct the literature search and screening process.

**Determining the disturbance and nutrient scenarios**

We classified studies into one of four categories/scenarios based on the level of disturbance (low/high) and nutrient availability (low/high) relative to the ecosystem of study (Appendix S2: Table S1). We selected this framework to enable categorization across ecosystems with varying disturbance and nutrient levels. Disturbance was defined based on the authors’ descriptions of the study design and system, typically involving a harmful environmental treatment (e.g., drought, low light conditions) imposed relative to natural conditions, which could potentially impair plant growth or induce mortality. We used the study’s description to determine the disturbance level; for example, if a paper compared how drought versus no-drought affected invasive and native plant roots, we considered the drought treatment as “High Disturbance.” Each study had to include information on the disturbance regime, type, and/or intensity. In cases where multiple levels of disturbance were provided (e.g., 4 levels of increasing drought stress), we selected the highest level of disturbance to represent “High Disturbance.” To qualify as “Low Disturbance”, the study had to explicitly state that the disturbance had been removed or that there was no disturbance in the experiment and/or field study.

Nutrient availability was defined based on soil nutrient levels described by the authors of the original study. To be included in the meta-analysis, studies had to clearly characterize an experimental treatment as having high or low nutrient levels. For example, a study that had a soil nutrient addition (e.g., ammonium, nitrate, or fertilizer addition) treatment was categorized as “High Nutrient” availability. This was also the same for the low nutrient papers: the authors had to explicitly state that the experiment was intended to evaluate how low soil nutrients affected native and invasive plant root traits. In most cases, these studies used low-quality soil or carbon amendments to immobilize soil nutrients to create poor-resource conditions.

The other inclusion criteria that were used to determine if a study was eligible include the following: (i) all papers had to compare invasive and native root traits within a specified ecological context of disturbance and nutrient availability; (ii) for papers that provided more than one invasive species, we treated each one as a separate case study; if the paper compared a community of invasive and native plants along with individual species data, we only used the community results; (iii) the study had to provide quantitative and/or report statistics on how root traits differed between native and invasive plants; (iv) the data from the study had to be from an original research article. Modeling, review, or meta-analytic papers were not included.

**Table S1.** Definitions of environmental scenarios used in our systematic review. We also provided an example of how the studies were characterized according to the disturbance and nutrient conditions. The ‘Root Traits’ column lists the types of root traits studied, along with the corresponding number of observations in parentheses. The ‘Final Count’ column indicates the number of invasive-native paired observations for each environmental scenario.

| **Scenario** | **Definition** | **Example study** | **Root traits** | **Final count** |
| --- | --- | --- | --- | --- |
| (a) Low Disturbance-High Nutrient | Characterized by a high abundance of soil nutrients, light, or water with low intensity disturbance events relating to anthropogenic impacts. | Nitrogen (N) addition without a disturbance treatment | N uptake (2), biomass (9), density (1), diameter (6), N content (1), root-to-shoot (5), specific root length (5), tissue density (2), surface area (1), N use efficiency (1) | 33 |
|  |  |  |  |  |
| (b) High Disturbance-High Nutrient | Defined as a high abundance of soil nutrients, light, or water, along with high intensity disturbance events. | Fertilizer addition crossed with a drought treatment | biomass (4), specific root length (2) | 6 |
|  |  |  |  |  |
| (b) Low Disturbance-Low Nutrient | Defined by limited soil nutrients, light, or water in a low disturbance environment. | Limited soil nutrients with no disturbance treatment | biomass (4), root-to-shoot (2), surface area (1) | 7 |
| (d) High Disturbance-Low Nutrient | Characterized by limited soil nutrients, light, or water, combined with a high level of disturbance. | Limited soil nutrients with a drought treatment | N uptake (1), biomass (5), diameter (1), N content (2), root-to-shoot (1), specific root length (2), tissue density (1) | 13 |
|  |  |  |  |  |


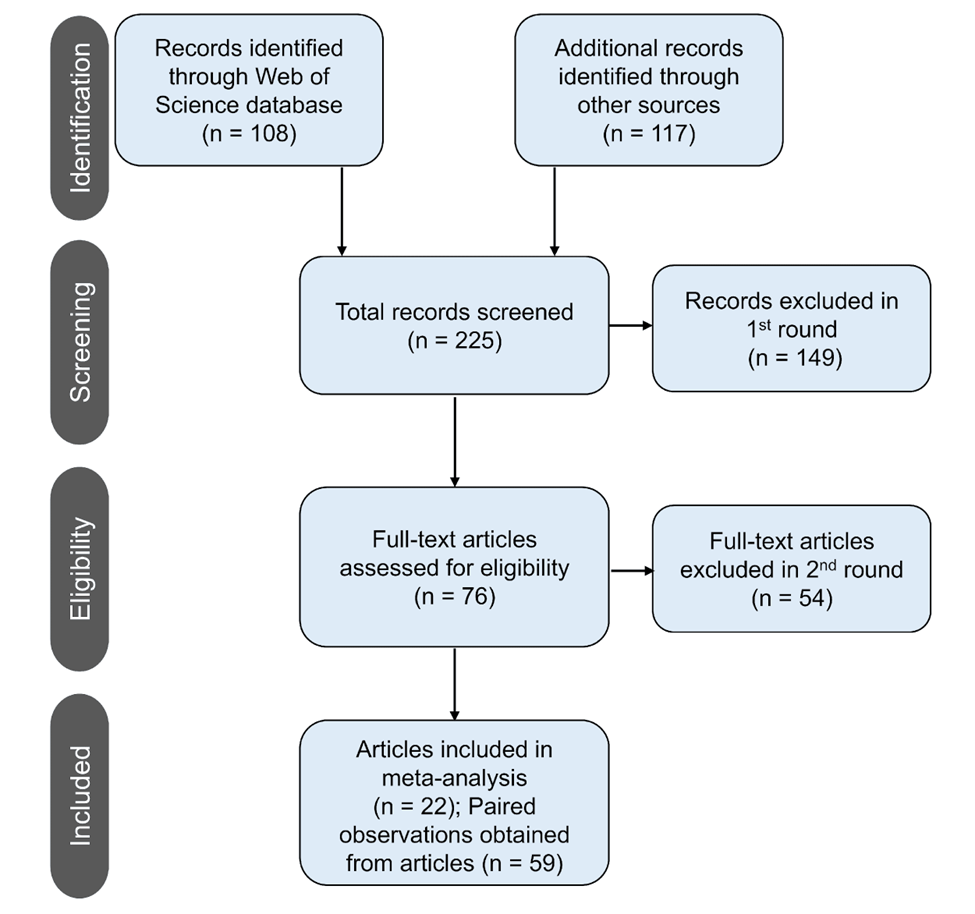


**Figure S1.** Our modified PRISMA flow schematic for the literature search and systematic review (Moher et al. 2009). During each step of the literature search, we recorded the total number of articles screened.

**References**

Moher, D., Liberati, A., Tetzlaff, J., & Altman, D. G. (2009). Preferred Reporting Items for Systematic Reviews and Meta-Analyses: The PRISMA Statement. PLoS Med, 6(7), e1000097–e1000097.
